# Prediction of Relevant Training Control Parameters at Individual Anaerobic Threshold without Blood Lactate Measurement

**DOI:** 10.3390/ijerph20054641

**Published:** 2023-03-06

**Authors:** Claudia Römer, Bernd Wolfarth

**Affiliations:** Department of Sports Medicine, Charité—Universitätsmedizin Berlin, Humboldt-University of Berlin, 10117 Berlin, Germany

**Keywords:** performance diagnostics, blood lactate, individual anaerobic threshold, training recommendation

## Abstract

Background: Active exercise therapy plays an essential role in tackling the global burden of obesity. Optimizing recommendations in individual training therapy requires that the essential parameters heart rate HR(IAT) and work load (W/kg(IAT) at individual anaerobic threshold (IAT) are known. Performance diagnostics with blood lactate is one of the most established methods for these kinds of diagnostics, yet it is also time consuming and expensive. Methods: To establish a regression model which allows HR(IAT) and (W/kg(IAT) to be predicted without measuring blood lactate, a total of 1234 performance protocols with blood lactate in cycle ergometry were analyzed. Multiple linear regression analyses were performed to predict the essential parameters (HR(IAT)) (W/kg(IAT)) by using routine parameters for ergometry without blood lactate. Results: HR(IAT) can be predicted with an RMSE of 8.77 bpm (*p* < 0.001), R^2^ = 0.799 (|R| = 0.798) without performing blood lactate diagnostics during cycle ergometry. In addition, it is possible to predict W/kg(IAT) with an RMSE (root mean square error) of 0.241 W/kg (*p* < 0.001), R^2^ = 0.897 (|R| = 0.897). Conclusions: It is possible to predict essential parameters for training management without measuring blood lactate. This model can easily be used in preventive medicine and results in an inexpensive yet better training management of the general population, which is essential for public health.

## 1. Introduction

Reluctance to undertake physical activity and obesity are associated with an increase in cardiovascular diseases, in particular in coronary heart disease, diabetes mellitus and a higher level of inflammation [1,2,3]. Research has demonstrated that engaging in regular physical activity leads to a reduction in both morbidity and mortality rates [4,5]. As our society continues to face an increasing burden of disease from conditions such as diabetes mellitus, arterial hypertension, and obesity, the cost of treating these cardiovascular diseases will also become a growing financial strain on the healthcare system in the future [6,7,8,9,10,11]. Especially, during the COVID-19 pandemic, regular exercising decreased significantly [12]. Consequences may be not only increasing obesity and cardiovascular diseases but also mental health conditions [13,14]. The WHO Guidelines recommend regular activity (150–300 min per week of moderate intensity, or 150 min per week of intensive physical activity) [15]. In order to achieve comprehensive prevention, simple and inexpensive training recommendations and the prescription of physical activity are required [16]. Optimizing training intensity recommendations in cardiopulmonary training requires that the essential parameters heart rate (HR) and training load (W/kg) at individual anaerobic threshold (IAT) are known [17]. It is necessary to define individual training parameters, as several studies have confirmed that training adherence depends on training intensity [18,19]. Adherence to physical activity is one of the most relevant factors to better health [20]. Realization of exercise recommendations by health workers is reported to be insufficient [21,22], which might be caused by the lack of personalized trainings programs. Overexertion, defined as the transition from the aerobic to the anaerobic metabolism [23], may therefore reduce training adherence. To perform optimal training, knowing the heart rate at the individual anaerobic threshold is essential for better training control. Measuring these important parameters (HR(IAT) and W/kg(IAT)) is largely limited to competitive athletes in dedicated sports medicine centers. Performance diagnostics with blood lactate is one of the most established methods for these kinds of diagnostics [24,25], but it is time- and cost-intensive [26].

The prediction of IAT was early performed by Conconi et al. by determining heart rate threshold which shows a high correlation in runners [27]. There are only a few studies using linear regression models to predict anaerobic threshold on cycling ergometry in the general population. Mostly athletes were examined, and the number of examined subjects is low [27,28,29,30]. Simple methods for measuring performance are crucial for allowing the general population access to the appropriate training parameters, particularly for individuals who are new to sports. There is a lack of studies examining prediction models for essential training parameters in the general population using non-invasive methods [31,32,33,34]. The aim of this study was to assess HR(IAT) and W/kg(IAT) by linear regression models to establish an easy access training recommendation for the general population, as physical inactivity is an important predictor of mortality [35].

## 2. Methods

In this study, a retrospective analysis was performed. Secondary data of the Sports Medicine Institute of the University Medical Center Charité Berlin were analyzed for the prediction of HR(IAT) and W/kg(IAT) without lactate measurement. All of the ergometry protocols conducted between 2015 and 2017 were obtained from the institutional sports medicine information system. Patients who were not included in the study were excluded for specific reasons: For the present analysis, the following inclusion criteria were applied: patients (I) with missing lactate data, (II) with missing heart rate data, and (III) with insufficient protocols and implausible data. Exclusion criteria were cardio-pulmonary and musculoskeletal diseases. The study was conducted in accordance with the Declaration of Helsinki and with the approval of the local ethics committee of Humboldt University Berlin.

### 2.1. Peak Performance Test

The performance test on the cycle ergometer started at 50 Watt (W) and was raised in 25 W steps after 3 min. Resting heart rate, blood pressure and blood lactate were measured before the lactate step test was initiated. During the test, heart rate was continuously measured by electrocardiogram. Blood pressure, blood lactate and RPE (rate of perceived exertion) were measured in the last thirty seconds of each step. Determination of lactate threshold (LT = first significant increase in blood lactate during exercise test starting from the resting lactate values) and individual lactate threshold (IAT = second significant increase in blood lactate and transition from aerobic to anaerobic metabolism) were assessed using the method of Dickhuth et al. [36].

### 2.2. Statistical Analysis

The Kolmogorov–Smirnov test was used to determine whether the continuous variables were normally distributed, and a descriptive analysis was carried out. The power per kilogram body weight at the individual anaerobic threshold (W/kg(IAT)) was used as a measure of individual physical performance. The Pearson correlation coefficient and root mean square error (RMSE) were used to assess correlation. A two-sided significance level of α = 0.001 was set as the threshold for determining statistical significance. Before performing multiple regression analysis of HR (IAT), all parameters were checked individually for their respective correlation and linear regression with a very high level of significance *p* < 0.001. Descriptive analysis was performed and is shown in Table 1. Minimum, mean and maximum HR and HR after one-, three- and five-minutes post-workout were examined. All parameters with a significance level *p* > 0.001 were removed. All statistical analyses were performed using the SPSS software (IBM Corp. Released 2016. IBM SPSS Statistics for Windows, Version 25.0. Armonk, NY, USA: IBM Corp.) and Matlab (MATLAB and Statistics Toolbox Release 2022b, The MathWorks, Inc., Natick, MA, USA).

#### Study Population

The population consisted of 188 competitive athletes (football, handball, athletics, volleyball, etc.), 226 prevention and rehabilitation athletes (with various chronic diseases, e.g., orthopedic, rheumatological or other autoimmune diseases) and 820 recreational athletes. None of the athletes had known coronary artery disease or heart failure. A total of 579 had a BMI greater than 25. Overall, 141 individuals of the 226 prevention and rehabilitation athletes had a BMI greater than 25, 52 individuals in this subgroup had a BMI greater than 30, and 13 individuals had a BMI greater than 35. Descriptive analysis is shown in Table 1.

## 3. Results

We performed multiple linear regression analyses for both HR(IAT) and W/kg(IAT) using personal parameters such as gender, age, height and weight as well as performance measurements such as heart rate and power as input parameters.

After each multiple regression analysis, we removed one parameter with the highest *p*-value until the desired significance level of *p* < 0.001 was met by all remaining input parameters.

**Figure 1:**
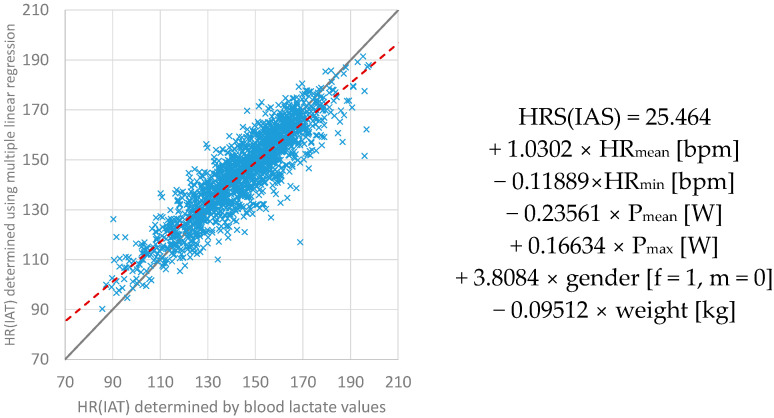
The comparison between HR(IAT) values on the horizontal axis determined by means of blood lactate values and the HR(IAT) values on the vertical axis determined by means of multiple linear regression.

After completing this process, the following input parameters are included in the multiple linear regression analysis for determining the HR(IAT); see equation in Figure 1: gender; weight; mean power (P_mean_); maximum power (P_max_); mean HR (HR_mean_); and minimal HR (HR_min_). Using these parameters in multiple linear regression, the determination of HR(IAT) is possible with an RMSE = 8.77 bpm. The adjusted R-squared is 0.798.

**Figure 2:**
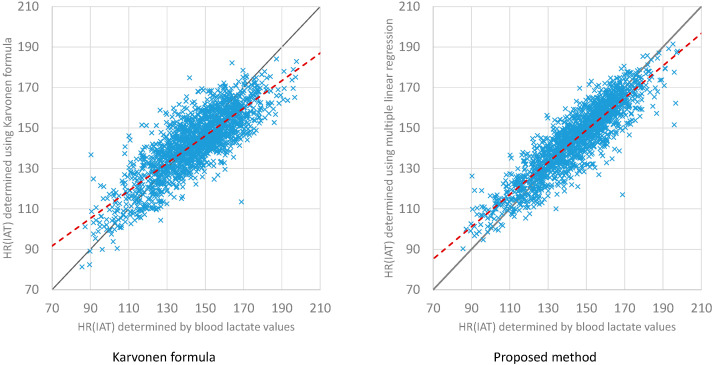
Comparison between regression model and Karvonen formula.

The proposed linear regression model for determining HR at IAT was compared to the Karvonen formula (Figure 2). The proposed method shows a lower RMSE (8.77 bpm) than the Karvonen formula (RMSE of 11.2 bpm), and HR determination at IAT is more exact using linear regression.

**Figure 3:**
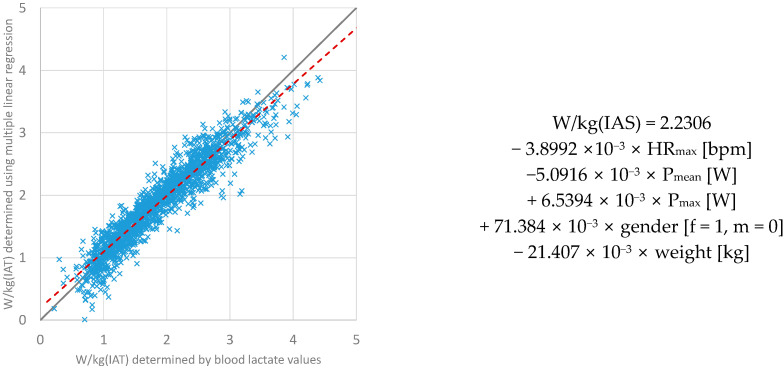
Impact of the individual input parameters on the W/kg(IAT).

The essential parameter W/kg(IAT), which is especially important for determining changes in performance, was also examined. As explained above, the respective input parameters were iteratively removed unless they met a level of significance *p* < 0.001. This includes the removal of the heart rate recovery (HRR = HRmax-HR after 5 min of recovery) parameter, as its significance level was *p* = 0.057. As a result, only the following four parameters were included for multiple linear regression analysis to determine W/kg(IAT): gender; body weight (kg); mean power (P_mean_); maximum power (P_max_); maximum HR (HR_max_). Using these parameters in multiple linear regression (Figure 3), the determination of W/kg(IAT) is possible with a root mean square error, RMSE = 0.241 W/kg. The adjusted R-squared was 0.897. Figure 3 shows the comparison between W/kg(IAT) values on the horizontal axis determined by means of blood lactate values and the W/kg(IAT) values on the vertical axis determined by means of multiple linear regression.

**Figure 4:**
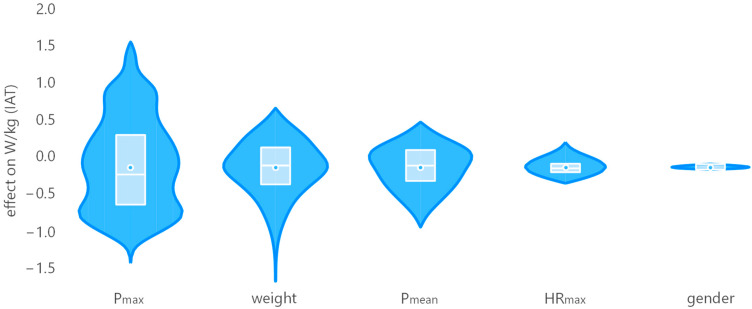
Effect plot for individual input parameters on W/kg(IAT) with parameter values normalized to a mean of zero for better comparison.

To better understand the impact of individual input parameters on the W/kg(IAT), we have visualized the regression parameters using an effect plot in Figure 4. For this, we multiplied the weights of the formula in Figure 3 with the actual values in our database. The latter are normalized by subtracting their respective mean values, as this offset is already modeled in linear regressions, in our case 2.2306 W/kg.

## 4. Discussion

This retrospective analysis of this dataset was examined to predict heart rate at IAT as well as training load (W/kg) at IAT without measuring blood lactate values for cycle ergometry. Both heart rate and the number of watts at the individual anaerobic threshold are essential parameters for training control. These parameters are currently best determined via blood lactate diagnostics during ergometry performance testing. A total of 1234 performance protocols with blood lactate in cycle ergometry were analyzed. Multiple linear regression analyses were performed to predict the essential parameters heart rate at individual anaerobic threshold (HR(IAT)) and workload at individual anaerobic threshold (W/kg(IAT)) by using routine parameters for ergometry without blood lactate. HR(IAT) can be predicted with a root mean square error, RMSE of 8.77 bpm (*p* < 0.001). The intention of this regression model is the acceleration of preventive medicine by using every ergometry to compile an individual training recommendation in primary and secondary prevention. At once, the greatest challenge and the utmost benefit is a continuous training adherence. To avoid overexertion, knowing the individual anaerobic threshold is necessary. This applies for preventive medicine as well as for pre-habilitation to meet the proposed exercise recommendation of 150–300 min per week by the WHO. Future work implies to supervise pre-habilitation patients with the recommended regression model, as standard cycle ergometry can be performed by every general practitioner, and patients can be examined close to the place of residence.

There is a need for more research in preventive medicine that focuses on developing better preventive training control methods for the general population. The main leverage point is the prevention of cardiovascular diseases, which is one of the most causes of morbidity and mortality. Furthermore, as societies are becoming older, frailty amongst the elderly population is a growing financial burden [37]. Increasing frailty goes in line with a decrease in quality of life. Regular physical activity can reduce frailty [38], and research has also shown an improvement for quality of life [39]. This challenge for health systems needed to be addressed by establishing easy access methods for training control parameters and training programs for the main population.

Although lactate performance diagnostics is a well-established method for recording performance, methodological errors must be considered. Due to constantly increasing blood lactate values and only intermittently measured lactate values by using the capillary blood of the earlobe, measurement inaccuracy must be assumed. Furthermore, certain nutritional methods (e.g., low-carb) are associated with an altered lactate curve [40]. Due to glycogen depletion, incorrect low blood lactate is measured, which can lead to a misinterpretation of the lactate curve. A large number of studies examined different threshold models, whereby an exact determination of the aerobic and anaerobic threshold is better to be regarded as an aerobic and anaerobic transition [41,42]. The earlier assumption of fixed aerobic and anaerobic thresholds soon showed individual differences in further investigations and the need to consider individual threshold methods. Despite these challenges of metabolic threshold models, the lactate determination for training recommendation was established as daily routine in contrast to other methods, in the last decades. The cardio-pulmonary exercise test (CPET), which is applied to determine VO2max and ventilatory thresholds, is also a method of assessing an individual’s physical fitness. For the collection of respiratory and metabolic parameters, this is a complex measurement, and expensive equipment with regular calibration is needed. This method is significantly more time-consuming, requires special trained nurses or sport scientists, and is therefore primarily reserved for patients with cardiac and pulmonary diseases. The RPE and the walking test are simple methods to avoid overexertion in preventive and recreational sports. Nevertheless, the application of RPE is difficult for people who are inexperienced in sports and can easily lead to overexertion or unchallenged activity. In preventive medical examination, an individual training recommendation is increasingly demanded by patients.

It could be shown that the lactate accumulation shows inter-individual differences [43,44,45], and fixed submaximal threshold concepts (of 2 mmol/and 4 mmol/l) should not be applied for individual training recommendations. The lactate concentration in the aerobic–anaerobic transition range is also dependent on muscle recruitment in different movement patterns [46,47]. Individual training recommendations should therefore be specific to the sport. Several studies have been able to prove the training effect of exercise based on the individual anaerobic threshold. The determination of the HR(IAT) during the ergometry without lactate diagnostics can be used for recommendations of basic endurance and interval training and for prevention programs.

Lactate measurement examination is an expensive, as lactate measurement equipment and special trained nurses are required, and time-consuming examination and has until now rarely been covered by health insurance companies. Depending on the individual, the test takes forty to fifty minutes, including warm-up, measuring resting heart rate and recovery time in the end. In various studies, the lactate transition range and the maximum lactate steady state showed a connection with hormonal and immunological changes, which at least supports the assumption of an upper anaerobic threshold [48,49,50]. Therefore, the individual anaerobic threshold should be considered when making training recommendations for the general population, since long-term training with a disproportionate increase in lactate can lead to training non-adherence and vulnerability for infections or injuries, thus bringing the known advantages of regular physical activity [49,50]. Our regression model allows a good prediction of HR(IAT) with an RSME 8.77 bpm and a prediction of W/kg(IAT) with a deviation of 0.241 W/kg. Shen et al. examined the velocity at lactate threshold on a treadmill by using several prediction models with different heart rates [31]. As with the data in this study, age was not a significant parameter and was excluded in the regression models. However, body mass index was excluded [31], and this study only included body weight for predicting W/kg(IAT). Interestingly, women seem to show a slightly higher W/kg unless body height is considered to be negative, in which case these effects neutralize each other. Differences between the results of Shen et al. also might be attributed to different physical activity on a treadmill and a cycle ergometry [31]. Sport-specific differences for HR(IAT) and W/kg(IAT) needed to be considered [51,52,53], and further research on regression models for running and rowing should be addressed in future studies.

The exclusion of heart rate recovery for the prediction of W/kg(IAT) was justified by not meeting the significance level of *p* < 0.001. This suggests that HRR may not be a singular predictor for evaluating physical fitness [54]. As research results are inconclusive and the evidence is weak [55,56,57], further research should be performed in larger studies. However, HRR should be recorded as a longitudinal parameter [54], since changes in HRR showed good results in recognizing cardiopulmonary diseases [58,59]. In this context, HRR is an essential parameter, which should be monitored regularly to detect changes in autonomic function [60].

The Karvonen formula is mainly used for training control in popular sports. The Karvonen formula uses the heart rate reserve, and it requires that the maximum heart rate and resting heart rate are determined to apply the formula [61]. By multiplication with a fitness level factor (0.8 for athletes; 0.6 for recreational athletes; and 0.3 for untrained people), the heart rate at the anaerobic threshold can be calculated [61]. The Karvonen formula was applied to the examined measurement protocol results in this study. The results of using the Karvonen formula with a factor of 0.7, due to the predominantly athletic clientele of the sports medicine university outpatient clinic, are shown in Figure 2. In comparison to the measured heart rate at IAT, the scatter diagram reveals a good correlation with a higher RMSE of 11.2 bpm in comparison to the regression model of this study (RMSE 8.77 bpm). The shape of the curve indicates an overestimation of the low values and an underestimation of the high HR values at the IAT. The Karvonen formula also uses resting HR and maximum heart rate to determine HR(IAT). Both heart rates are individual values, and maximum heart rate is especially difficult to determine for the general population, especially as maximum heart rate changes with age [62,63,64]. Thus, an initial determination of maximum heart rate is also required for the Karvonen formula and should be acquired under medical supervision, especially for individuals > 35 years to cardiovascular adverse events. Due to the improved prediction based on a regression model determined in this study, we recommend cycle ergometry in medical supervision with the regression model identified in Figure 1.

In preventive medicine, ergometry is also recommended for every sports beginner and returner over the age of 40 (for men) and over 55 (for women), according to the German guideline for preventive medical check-ups in sports. In contrast to lactate performance diagnostics, ergometry can be carried out by almost any general practitioner or as part of an occupational medical examination. However, an individual training recommendation is usually only given by sports physicians, since a respective specialization for individual training advice is missing. Due to the increasing number of cardiovascular events, obesity and an increasingly aging population, there is a health gap to reach the general population with individual training recommendations and to examine the full scope of preventive medicine. The proposed regression model differs from other studies with a significantly higher number of study protocols examined in a heterogeneous population [27,28]. In addition, further research should examine whether shorter exercise tests, such as the 6-MWT (6-min walking test), can be used for a regression model prediction of essential parameters for training control [65]. Studies in obese individuals demonstrated promising potential to assess individual respiratory threshold [65,66,67]. Especially obese and older subjects or individuals with other disabilities which rule out cycle ergometry might benefit [65]. Thus, a regression model for cycle ergometry with a shortened protocol should be addressed in future studies, as these shorter tests can be performed more regularly to examine training improvement and address the changed HR(IAT) after consistent training [66]. Considering the results of this retrospective study, we recommend the output of a training program with an individual training heart rate at IAT and watt range at IAT, provided after every check-up examination using cycle ergometry in medical supervision, including the recommendation of the WHO [15].

### Future Work

As it is known that also children and adolescent obesity has been continuously rising during the last few years [68], there is a need to find approaches for physical activity in these age groups, since chronic diseases will start in early ages and will have a huge impact on GNP. Further studies are necessary to establish regression models for HF(IAT) for adolescents to teach them a healthy and adequate regular exercise program with a potentially better exercise adherence, since exercise adherence is one of the most encouraging parameters for health [20]. These exercise programs should be established in schools under supervision and with regular physical examinations. Furthermore, research has shown that pre-habilitation can be a relevant benefit prior to chemotherapy or extensive operations [69,70]. As neoadjuvant chemotherapy is associated with decreasing aerobic endurance [70,71], there is a need for easy access training therapy not only in primary but also in secondary and tertiary prevention. Measuring HF(IAT) at routine secondary and tertiary preventive examinations may improve exercise adherence; further research in these subgroups is necessary.

Further goals of these examinations are an establishment of pre-habilitation offers close to home, besides the expansion of the preventive individual training recommendations for the general population. An individual training recommendation for pre-habilitation could therefore be made directly by the attending general practitioner or cardiologist. A gap in care, of mostly only a few sports medicine offers, could thus be closed. Further examinations with other ergometer types (rowing ergometer, elliptical) are planned in order to enable a conversion of HR(IAT) and different ergometer types in prevention and pre-habilitation.

## 5. Limitations

Incorrect entries during the manual transmission of the lactate values must be considered. These were minimized in advance by means of a plausibility check of the entire data set. The sample size in this study is appropriate for generating a valuable prediction in comparison to other studies [31,72]. The age and gender distribution may vary in comparison to the general population, as the examined population includes more physically active individuals, especially in the younger age groups. Furthermore, a heart rate deviation of 8.77 bpm is not appropriate for athletes in professional sports, although a blood lactate test or cardiopulmonary exercise test (CPET) is still recommended for this clientele. This regression model is suitable for cardiorespiratory endurance sports. It should be noticed that it is not applicable for resistance or interval training; individual training recommendations for these kinds of training should be considered.

At the same time, ergometry offers a simple and inexpensive measuring method that can be performed in the outpatient and inpatient sector and represents a suitable procedure for popular sports and preventive medicine to monitor cardiorespiratory training.

## 6. Conclusions

In conclusion, it is possible to derive relevant parameters for training control after a standard cycle ergometry without performing a blood lactate test by using regression models to predict HR(IAT) and W/kg(IAT) for the general population. This enables training control without blood lactate diagnostics or CPET and does achieve enormous time and financial savings for active exercise therapy as well as for preventive and rehabilitative medicine. Regular individual test repetition allows the consideration of short-term training adaption and supports continuous training progress.

## Figures and Tables

**Table 1:** Descriptive characteristics of female and male individuals.

		Female (*n* = 469)	Male (*n* = 765)
		Mean ± SD	Mean ± SD
Age (years)		48.24 ± 19.36	46.61 ± 18.60
Height (cm)		166.25 ± 7.79	180.33 ± 7.93
Weight (kg)		67.12 ± 12.37	84.77 ± 13.53
Pmax/kg (W/kg)		2.16 ± 0.91	2.78 ± 1.02
P_mean_ (W)	Mean power	89.85 ± 35.44	141.19 ± 48.68
HR_min_	Minimum HR	73.30 ± 12.53	70.38 ± 12.41
HR_mean_	Mean HR	124.00 ± 16.83	123.32 ± 15.94
HR_max_	Maximum HR	166.99 ± 20.93	170.13 ± 20.78
HR_pw1_	HR 1 min post workout	142.43 ± 22.63	144.85 ± 20.04
HR_pw3_	HR 3 min post workout	115.66 ± 20.04	119.26 ± 17.44
HR_pw5_	HR 5 min post workout	103.80 ± 18.57	108.59 ± 16.60
HRR	delta HR_max_ − HR_pw5_	63.19 ± 14.04	61.54 ± 15.02
P/kg(IAT) (W/kg)		1.58 ± 0.64	1.98 ± 0.77
HR(IAT)		143.99 ± 18.66	141.12 ± 19.46

## Data Availability

The data presented in this study are available on request from the corresponding author. The data are not publicly available due to data privacy regulations.

## Abbreviations

CPET	cardiopulmonary exercise test
HR	heart rate
HR (IAT)	heart rate at individual anaerobic threshold
HR_max_	maximum heart rate
HRR	heart rate recovery = HRmax-HR after 5 min of recovery
P_max_	maximum power output
W/kg(IAT)	Watt per kg at individual anaerobic threshold
RMSE	root mean square error
W	Watt
